# Changes in Childhood Atopic Dermatitis Incidence and Risk Factors Over Time: Results From Two German Birth Cohorts

**DOI:** 10.1111/cea.70066

**Published:** 2025-04-28

**Authors:** Zhuoxin Peng, Linda P. Siziba, Hermann Brenner, Deborah Wernecke, Dietrich Rothenbacher, Jon Genuneit

**Affiliations:** ^1^ Pediatric Epidemiology, Department of Pediatrics Medical Faculty, Leipzig University Leipzig Germany; ^2^ Division of Clinical Epidemiology and Aging Research German Cancer Research Center Heidelberg Germany; ^3^ Institute of Epidemiology and Medical Biometry Ulm University Ulm Germany; ^4^ German Center for Child and Adolescent Health (DZKJ), Partner Site Ulm Ulm Germany; ^5^ German Center for Child and Adolescent Health (DZKJ), Partner Site Leipzig Leipzig Germany

**Keywords:** atopic dermatitis, cumulative incidence, environment, microbiota, risk factors

## Abstract

**Background:**

Atopic dermatitis (AD) is a common allergic skin disease. We aimed to assess the secular changes in the cumulative incidence of childhood AD and its risk factors over a decade.

**Methods:**

We used data from two methodologically similar cohort studies in Ulm, Germany, the Ulm Birth Cohort Study (UBCS, recruited in 2000/2001) and the Ulm SPATZ Health Study (recruited in 2012/2013). The cumulative incidences of AD as reported by their family physicians and parents up to the age of 4 years were compared by log‐rank test across the two cohorts, using propensity score–based weighting to control confounders. We fitted multivariable Cox regression models to estimate hazard ratios and 95% confidence intervals (CIs) for the factors associated with the occurrence of physician‐ and parent‐reported AD and compared the results between the two cohorts.

**Results:**

The 4‐year cumulative incidence (95% confidence interval) of physician‐reported AD (27.4% (24.4%–30.5%) in UBCS [2000/2001] vs. 26.4% (22.8%–30.2%) in SPATZ [2012/2013], *p* = 0.728) and parent‐reported AD (14.5% (12.2%–17.0%) in UBCS [2000/2001] vs. 16.7% (14.0%–19.7%) in SPATZ [2012/2013], *p* = 0.211) remained stable between the two cohorts after propensity score–based weighting. We observed the changes in the association between AD and certain risk factors (e.g., family history of AD and infantile antibiotic use) over the decade, but the results need to be interpreted with caution due to the limited sample size, relatively high attrition rate and demographic differences between the two cohorts.

**Conclusions:**

Over the decade, childhood AD incidence remained stable. Further studies are needed to verify whether there is a growing importance of environmental and microbiota‐related factors for AD development over time.

## Graphical abstract

Cumulative incidences of childhood atopic dermatitis (AD) up to the age of 4 and the risk factors were assessed and compared between two methodologically similar birth cohorts in Ulm, Germany: UBCS (recruited in 2000/2001) and SPATZ (recruited in 2012/2013). Over the decade, childhood AD incidence remained stable, but there may have been a growing importance of environmental and microbiota‐related factors influencing AD development, that is, having older siblings at home and infantile antibiotic use.

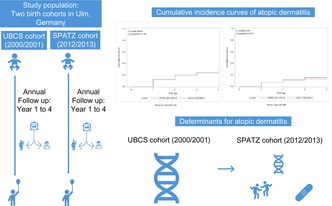

**Summary**

- Cumulative incidences of AD by the age of 4 remained stable over the decade (2000/2001–2012/2013).
- We observed changes in the association between AD and certain risk factors over the decade.
- There is a disagreement on risk factors for AD by physicians' or parents' reports.

## 1 Introduction

Atopic dermatitis (AD) is a common inflammatory skin disease in childhood and is often associated with other atopic diseases (e.g., asthma and atopic rhinitis) in later life [1]. The estimated worldwide prevalence of current AD for children aged 6–7 years was 7.9% according to The International Study of Asthma and Allergies in Childhood (ISAAC) Phase Three (1999–2004); the prevalence was highest in Ecuador (22.5%) and lowest in India (0.9%) [2]. A more recent global survey, the Global Asthma Network (GAN) Phase I (2015–2020), reported a small increase in AD prevalence over time among children aged 6–7 years (1.21% in 10 years) [3].

Besides geographical regions, the prevalence of atopic dermatitis varies among different age groups. The incidence of AD is highest during the first 18 months of life and drops after adolescence [4]. Family history of atopic diseases, socioeconomic status, lifestyle and environmental factors are also known determinants of AD development [5]. It is consistently reported that having a family history of atopic diseases, including AD, asthma and atopic rhinitis, will increase the risk of developing AD among children [6]. The hygiene hypothesis suggests that environmental exposure to certain microorganisms in early life may foster the development of the immune system and a lack of such exposure may trigger the occurrence of atopic dermatitis [7]. Higher socioeconomic status often leads to decreased exposure to microorganisms and subsequently results in an increased risk of AD development [8].

Although previous studies have provided important information on the secular trend of childhood AD incidence and prevalence, data on secular changes of the longitudinal course of atopic dermatitis as well as the changes in the factors associated with AD development in early life is still rare. Using data from two methodologically similar cohorts in Germany recruited 12 years apart, the Ulm Birth Cohort Study (UBCS) and the Ulm SPATZ Health Study, we aimed to compare the cumulative incidence of childhood atopic dermatitis up to the age of 4 years in the cohort study. Additionally, we assessed the changes in factors associated with the occurrence of atopic dermatitis between these two birth cohorts.

## 2 Methods

### 2.1 Cohort Overview

UBCS and SPATZ are two birth cohort studies with similar methodologies, which recruited infants and their mothers from the general population after birth from 11/2000–11/2001 to 04/2012–05/2013, respectively, in the University Medical Center Ulm, Ulm, Germany. This hospital was the only maternity hospital in Ulm during the recruitment periods. Exclusion criteria were outpatient delivery, maternal age below 18 years, immediate transfer of the newborn or mother to intensive care post‐delivery or inadequate proficiency in the German language. At baseline, the UBCS (2000/2001) included 1066 mothers and 1090 newborns; the SPATZ (2012/2013) cohort included 970 mothers and 1006 newborns. We collected maternal socioeconomic and health information at baseline shortly after the delivery in both cohorts. Then we followed up with the mothers and infants annually and in parallel asked the children's primary care physicians (some of them are also specialised in paediatrics) to complete questionnaires for health information of children. Both the UBCS (2000/2001) and the SPATZ (2012/2013) study were approved by the ethics board of Ulm University (UBCS: No. 98/2000; SPATZ: No. 311/11). More details regarding the two birth cohorts have been published elsewhere [9].

### 2.2 Outcome and Covariates

The outcomes in this study are physician‐reported and parent‐reported AD diagnosis status (yes/no) of children based on the separately self‐administered annual physicians' and parents' questionnaires at 1‐, 2‐, 3‐ and 4‐year follow‐up in the UBCS (2000/2001) and SPATZ (2012/2013) cohort, respectively [10]. The question regarding children's AD diagnosis in the UBCS (2000/2001) and SPATZ (2012/2013) questionnaires was ‘Has one of the following diseases: neurodermatitis, endogenous eczema, or atopic dermatitis, been diagnosed by a physician until now?’ The same question appeared in both physicians' and parents' questionnaires and we extracted the information on physician‐reported and parent‐reported AD based on the answers from physicians and/or parents to this question. Both reports were for a doctor's diagnosis although not necessarily made by the caring and responding paediatricians themselves. We only included the participants who at least completed the questionnaire for the first‐year follow‐up in the analyses, as the AD information was initially collected at the first‐year follow‐up. If a participant dropped out of the follow‐up later, he or she was censored at the year of the last follow‐up.

We extracted the information on maternal and children's characteristics as covariates potentially associated with the development of AD from the baseline and follow‐up questionnaires in both cohorts. The family characteristics include maternal age at baseline (< 30 years old, ≥ 30 and < 35 years old, ≥ 35 years old), mothers' education level (schooling > 11 years: yes vs. no), children's first‐degree relatives' (i.e., mothers, fathers and older siblings) history of AD at baseline, mother ever having a fever during pregnancy (yes vs. no), maternal smoking status during pregnancy (yes vs. no) and maternal BMI before pregnancy (> 25 vs. ≤ 25 kg/m^2^). The children's characteristics include sex, preterm birth (gestational age < 37 weeks, yes vs. no), delivery mode (c‐section vs. vaginal delivery), birth weight (< 2500 g, 2500–4000 g, > 4000 g), exclusive breastfeeding duration (< 4 months vs. ≥ 4 months), history of antibiotic use in the first year of life (yes vs. no) and having at least one older sibling (yes vs. no).

### 2.3 Statistical Analyses

We first compared the family and children's characteristics between the UBCS (2000/2001) and the SPATZ (2012/2013) cohort by chi‐square test. Then we calculated the crude 1‐, 2‐, 3‐ and 4‐year cumulative incidences of physician‐ and parent‐reported AD in the two cohorts. The distribution of the cumulative incidences was compared by log‐rank test across the two cohorts. In addition, we used a logistic regression model to calculate propensity scores (PS) for the two cohorts based on the family and children's characteristics as potential confounders suggested in the literature, that is, the maternal age at baseline, mothers' education level, children's first‐degree relatives' history of AD at baseline, mother ever having a fever during pregnancy, maternal smoking status during pregnancy, maternal BMI before pregnancy, children's sex, preterm birth, delivery mode, birth weight, exclusive breastfeeding duration, history of antibiotic use in the first year of life and having at least one older sibling or not. Using the PS‐weighted log‐rank test, we compared the distribution of cumulative incidences across the two cohorts again, in order to account for the influence of the difference in family and children's characteristics between the two cohorts.

We fitted multivariable Cox proportional hazards models to estimate hazard ratios (HRs) and 95% confidence intervals (CIs) for the factors associated with the occurrence of physician‐ and parent‐reported AD in each cohort and assessed whether the statistical significance of factors changed between the cohorts. The same set of maternal and children's characteristics above was included as covariates in the Cox proportional hazards models.

In addition, we performed three sensitivity analyses to validate the robustness of our results. First, we combined the physician‐ and parent‐reported AD diagnosis as the outcome: if either the physician or the parents report an AD diagnosis, then the child would be regarded as an AD case. Second, we performed a multiple imputation for the missing values in the data. We generated 20 imputed datasets with a fully conditional specification approach, using all the covariates above as predictors for missing values and analysed the pooled results. Third, as the AD diagnoses in the first year of life might overlap with other dermatoses of infancy, we only included the AD diagnoses from Year 2 as the outcome in the models.

All the statistical analyses were performed using SAS (version 9.4).

## 3 Results

**FIGURE 1:**
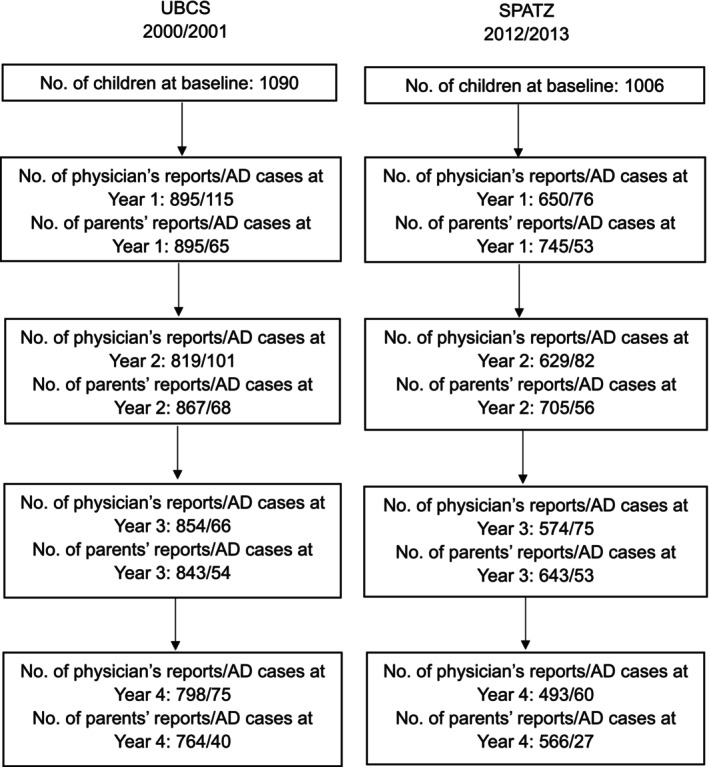
Flowchart of participant numbers from UBCS and SPATZ at baseline and each follow‐up.

The number of participants with completed parents' and physicians' questionnaires from the UBCS (baseline recruitment in the years 2000/2001) and the SPATZ (years 2012/2013, respectively) cohort and with information on AD diagnoses at each follow‐up is presented in Figure 1. A total of 895 children with physician/parent reports in UBCS (2000/2001) as well as 650 with physician reports and 745 with parent reports in SPATZ (2012/2013) were included in the trend analysis. We compared the child and parental characteristics between the UBCS (2000/2001) and SPATZ (2012/2013) cohort in Table 1. In comparison with the participants in the UBCS (2000/2001), mothers in SPATZ (2012/2013) were significantly older during pregnancy (aged under 30 years old: 26.5% in SPATZ vs. 37.2% in UBCS), had a higher education (57.4% in SPATZ vs. 36.5% in UBCS), were more likely to be overweight (34.7% in SPATZ vs. 28.7% in UBCS) and were less likely to have a fever during pregnancy (9.1% in SPATZ vs. 20.2% in UBCS) and to have smoked during pregnancy (7.2% in SPATZ vs. 14.8% in UBCS); children in SPATZ (2012/2013) study were more likely to have encountered a preterm birth (11.3% in SPATZ vs. 5.6% in UBCS), c‐section delivery (27.9% in SPATZ vs. 17.3% in UBCS) and to have a birth weight below 2500 g (9.0% in SPATZ vs. 3.1% in UBCS).

**TABLE 1:** Baseline characteristics of children and parents in the UBCS and SPATZ cohorts.

	UBCS (2000/2001)	SPATZ (2012/2013)	*p*
	*N* (%)	*N* (%)	
No. at baseline	1090 (100)	1006 (100)	
Family characteristics			
Maternal age during pregnancy			
< 30 years old	405 (37.2)	267 (26.5)	< 0.001*
≥ 30 years old and < 35 years old	412 (37.8)	428 (42.5)	
≥ 35 years old	273 (25.1)	311 (30.9)	
Maternal schooling > 11 years	398 (36.5)	577 (57.4)	< 0.001*
First‐degree relatives with AD	150 (13.8)	162 (16.3)	0.118
Maternal BMI > 25	313 (28.7)	349 (34.7)	0.003*
Mothers having a fever during pregnancy	219 (20.2)	90 (9.1)	< 0.001*
Maternal smoking during pregnancy	161 (14.8)	71 (7.2)	< 0.001*
Children's characteristics			
Male	551 (50.6)	522 (51.9)	0.553
Preterm birth	61 (5.6)	114 (11.3)	< 0.001*
C‐section delivery	189 (17.3)	281 (27.9)	< 0.001*
Birth weight			
< 2500 g	34 (3.1)	90 (9.0)	< 0.001*
≥ 2500 g and < 4000 g	940 (86.2)	851 (84.6)	
≥ 4000 g	116 (10.6)	65 (6.5)	
Having at least one older sibling	551 (50.6)	352 (48.2)	0.298
Antibiotic use in the first year of life	110 (14.4)	118 (18.2)	0.058
Exclusive breastfeeding > 4 months	476 (51.6)	355 (46.9)	0.057

Abbreviations: AD, atopic dermatitis; BMI, body mass index.

*p < 0.05.

**FIGURE 2:**
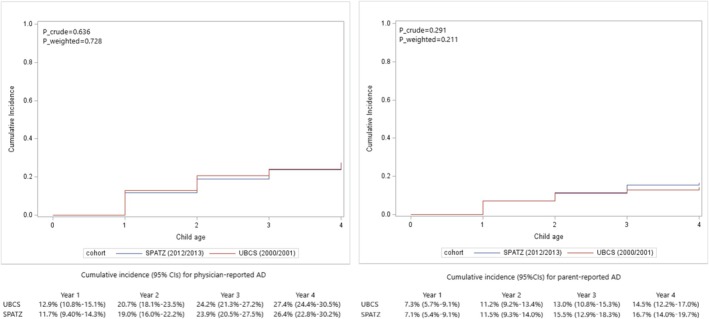
Overall cumulative incidence curves of physician‐ and parent‐reported AD in UBCS and SPATZ cohort from Year 1 to Year 4. P_crude is for the crude log‐rank test for comparing the cumulative incidence of AD between two cohorts; P_weighted is for the propensity score weighted log‐rank test for comparing the cumulative incidence of AD between two cohorts. The propensity score is based on the maternal age at baseline, mothers' education level, children's first‐degree relatives' history of AD at baseline, mothers ever having a fever during pregnancy, maternal smoking status during pregnancy, maternal BMI before pregnancy, children's sex, preterm birth, delivery mode, birth weight, exclusive breastfeeding duration, history of antibiotic use in the first year of life and having at least one older sibling or not. AD, atopic dermatitis; CIs, confidence intervals.

Figure 2 shows the overall cumulative incidence curves in the UBCS (2000/2001) and the SPATZ (2012/2013) cohort from Year 1 to Year 4. The 4‐year cumulative incidence of physician‐reported AD was 27.4% (95% CIs: 24.4%–30.5%) in UBCS (2000/2001) and 26.4% (95% CIs: 22.8%–30.2%) in SPATZ (2012/2013). The 4‐year cumulative incidence of parent‐reported AD was 14.5% (95% CIs: 12.2%–17.0%) in UBCS (2000/2001) and 16.7% (95% CIs: 14.0%–19.7%) in SPATZ (2012/2013). The log‐rank test showed that there was no significant difference between these two cohorts in the distribution of crude cumulative incidence of physician‐reported AD (*p* = 0.636) and parent‐reported AD (*p* = 0.291). The PS‐weighted log‐rank test revealed similar results (*P*_weighted_ = 0.728 for physician‐reported AD and *P*_weighted_ = 0.211 for parent‐reported AD).

When we combined the UBCS (2000/2001) and SPATZ (2012/2013) cohort (participants with AD information *n* = 1640 in total), Cox proportional hazards models in Table 2 showed that, after adjusting for multiple covariates and the cohorts, having a first‐degree relative with AD was associated with physician‐reported (HR: 1.41, 95% CIs: 1.08–1.84, *p* = 0.011) and parent‐reported AD (HR: 1.82, 95% CIs: 1.31–2.52, *p* < 0.001); whereas maternal smoking during pregnancy was just associated with parent‐reported AD (HR: 1.85, 95% CIs: 1.07–3.21, *p* = 0.029).

**TABLE 2:** Risk factors associated with physician‐ and parent‐reported AD in the combined cohorts of UBCS and SPATZ (*N* = 1640 participants in the complete case analysis).

	Physician‐reported AD		Parent‐reported AD	
	HR (95% CIs)^a^	*p*	HR (95% CIs)	*p*
Family characteristics				
Maternal age during pregnancy				
≥ 30 years old and < 35 years old	1		1	
< 30 years old	1.19 (0.90–1.57)	0.222	1.16 (0.81–1.66)	0.408
≥ 35 years old	1.01 (0.77–1.32)	0.956	0.92 (0.65–1.31)	0.652
Maternal schooling > 11 years				
No	1		1	
Yes	1.00 (0.79–1.27)	0.984	1.11 (0.81–1.52)	0.509
First‐degree relatives with AD				
No	1		1	
Yes	1.41 (1.08–1.84)	0.011*	1.82 (1.31–2.52)	< 0.001*
Maternal BMI > 25				
No	1		1	
Yes	0.90 (0.70–1.15)	0.395	0.85 (0.61–1.18)	0.337
Mothers having a fever during pregnancy				
No	1		1	
Yes	1.17 (0.88–1.57)	0.285	0.98 (0.66–1.46)	0.983
Maternal smoking during pregnancy				
No	1		1	
Yes	1.16 (0.72–1.86)	0.553	1.85 (1.07–3.21)	0.029*
Children's characteristics				
Sex				
Female	1		1	
Male	1.03 (0.82–1.28)	0.813	0.99 (0.75–1.32)	0.969
Preterm birth				
No	1		1	
Yes	0.72 (0.41–1.27)	0.261	0.66 (0.31–1.39)	0.271
C‐section delivery				
No	1		1	
Yes	1.13 (0.86–1.48)	0.389	0.87 (0.60–1.26)	0.454
Birth weight				
≥ 2500 g and < 4000 g	1		1	
< 2500	0.65 (0.32–1.33)	0.238	1.00 (0.43–2.34)	0.993
≥ 4000 g	0.97 (0.67–1.40)	0.866	1.24 (0.80–1.95)	0.339
Having at least one older sibling				
No	1		1	
Yes	0.86 (0.68–1.09)	0.212	0.92 (0.68–1.24)	0.572
Antibiotic use in the first year of life				
No	1		1	
Yes	1.30 (0.98–1.72)	0.071	1.23 (0.86–1.76)	0.261
Exclusive breastfeeding > 4 months				
No	1		1	
Yes	1.05 (0.84–1.32)	0.657	1.27 (0.94–1.71)	0.118

Abbreviations: AD, atopic dermatitis; BMI, body mass index; CIs, confidence intervals; HR, hazard ratio.

^a^ We fitted multivariable Cox proportional hazard models to estimate hazard ratios (HRs) and 95% confidence intervals (CIs) for AD, controlling for all the factors in the table and the cohorts (SPATZ vs. UBCS).

*P < 0.05.

The risk factors for AD development differed when we analysed the UBCS (2000/2001) and SPATZ (2012/2013) cohorts separately. As depicted in Table 3, Cox proportional hazards models showed that only having a first‐degree relative with AD (HR: 1.76, 95% CIs: 1.24–2.49, *p* = 0.002) was significantly associated with an increased risk of physician‐reported AD in UBCS (2000/2001). In SPATZ (2012/2013), having a first‐degree relative with AD was not statistically significantly associated with physician‐reported AD (yes vs. no, HR: 1.08, 95% CIs: 0.71–1.63, *p* = 0.723). Instead, having at least one older sibling (yes vs. no, HR: 0.65, 95% CIs: 0.45–0.93, *p* = 0.020) and antibiotic use in the first year of life (yes vs. no, HR: 1.51, 95% CIs: 1.02–2.24, *p* = 0.042) were significant factors associated with reduced and increased risk of physician‐reported AD, respectively.

**TABLE 3:** Risk factors associated with physician‐reported AD in the UBCS (*N* = 895) and SPATZ (*N* = 650) cohort.

	UBCS (2000/2001)		SPATZ (2012/2013)	
	HR (95% CIs)^a^	*p*	HR (95% CIs)	*p*
Family characteristics				
Maternal age during pregnancy				
≥ 30 years old and < 35 years old	1		1	
< 30 years old	1.08 (0.75–1.57)	0.672	1.41 (0.93–2.14)	0.102
≥ 35 years old	0.99 (0.70–1.42)	0.976	1.07 (0.71–1.59)	0.751
Maternal schooling > 11 years				
No	1		1	
Yes	0.97 (0.71–1.32)	0.838	1.03 (0.71–1.50)	0.868
First‐degree relatives with AD				
No	1		1	
Yes	1.76 (1.24–2.49)	0.002*	1.08 (0.71–1.63)	0.723
Maternal BMI > 25				
No	1		1	
Yes	0.98 (0.69–1.38)	0.888	0.77 (0.53–1.13)	0.180
Mothers having a fever during pregnancy				
No	1		1	
Yes	1.21 (0.85–1.71)	0.299	1.22 (0.72–2.05)	0.462
Maternal smoking during pregnancy				
No	1		1	
Yes	1.18 (0.67–2.08)	0.562	0.90 (0.36–2.28)	0.823
Children's characteristics				
Sex				
Female	1		1	
Male	0.91 (0.68–1.23)	0.554	1.17 (0.83–1.63)	0.374
Preterm birth				
No	1		1	
Yes	0.90 (0.41–1.97)	0.789	0.52 (0.23–1.18)	0.118
C‐section delivery				
No	1		1	
Yes	1.13 (0.75–1.69)	0.556	1.17 (0.80–1.71)	0.420
Birth weight				
≥ 2500 g and < 4000 g	1		1	
< 2500 g	0.36 (0.09–1.50)	0.160	1.00 (0.41–2.42)	0.999
≥ 4000 g	1.05 (0.67–1.64)	0.845	0.89 (0.47–1.68)	0.721
Having at least one older sibling				
No	1		1	
Yes	1.03 (0.74–1.42)	0.863	0.65 (0.45–0.93)	0.020*
Antibiotic use in the first year of life				
No	1		1	
Yes	1.13 (0.75–1.71)	0.558	1.51 (1.02–2.24)	0.042*
Exclusive breastfeeding > 4 months				
No	1		1	
Yes	0.96 (0.70–1.32)	0.788	1.13 (0.82–1.58)	0.455

Abbreviations: AD, atopic dermatitis; BMI, body mass index; CIs, confidence intervals; HR, hazard ratio.

^a^ We fitted multivariable Cox proportional hazard models to estimate hazard ratios (HRs) and 95% confidence intervals (CIs) for AD, controlling for all the factors in the table.

*p < 0.05.

Table 4 also indicates that only having a first‐degree relative with AD (HR: 1.93, 95% CIs: 1.21–3.10, *p* = 0.006) was significantly associated with an increased risk of parent‐reported AD in the UBCS (2000/2001). The factors associated with parent‐reported AD in SPATZ (2012/2013) were first‐degree relatives with AD (yes vs. no, HR: 1.64, 95% CIs: 1.03–2.62, *p* = 0.038) and antibiotic use in the first year of life (yes vs. no, HR: 1.75, 95% CIs: 1.11–2.78, *p* = 0.017).

**TABLE 4:** Risk factors associated with parent‐reported AD in UBCS (*n* = 895) and SPATZ (*N* = 745) cohort.

	UBCS (2000/2001)		SPATZ (2012/2013)	
	HR (95% CIs)^a^	*p*	HR (95% CIs)	*p*
Family characteristics				
Maternal age during pregnancy				
≥ 30 years old and < 35 years old	1		1	
< 30 years old	0.88 (0.54–1.44)	0.612	1.57 (0.93–2.64)	0.091
≥ 35 years old	0.75 (0.45–1.24)	0.258	1.18 (0.72–1.93)	0.521
Maternal schooling > 11 years				
No	1		1	
Yes	1.16 (0.76–1.76)	0.501	1.07 (0.68–1.70)	0.767
First‐degree relatives with AD				
No	1		1	
Yes	1.93 (1.21–3.10)	0.006*	1.64 (1.03–2.62)	0.038*
Maternal BMI > 25				
No	1		1	
Yes	0.88 (0.54–1.44)	0.614	0.87 (0.54–1.39)	0.551
Mothers having a fever during pregnancy				
No	1		1	
Yes	1.05 (0.64–1.72)	0.848	0.95 (0.48–1.85)	0.869
Maternal smoking during pregnancy				
No	1		1	
Yes	1.80 (0.91–3.57)	0.092	1.82 (0.71–4.70)	0.216
Children's characteristics				
Sex				
Female	1		1	
Male	0.81 (0.54–1.21)	0.294	1.21 (0.79–1.83)	0.385
Preterm birth				
No	1		1	
Yes	0.87 (0.27–2.79)	0.808	0.41 (0.15–1.17)	0.097
C‐section delivery				
No	1		1	
Yes	0.72 (0.38–1.36)	0.309	0.95 (0.59–1.54)	0.847
Birth weight				
≥ 2500 g and < 4000 g	1		1	
< 2500	N/A^b^	N/A	2.16 (0.82–5.70)	0.119
≥ 4000 g	1.16 (0.64–2.11)	0.624	1.44 (0.73–2.85)	0.295
Having at least one older sibling				
No	1		1	
Yes	0.86 (0.56–1.32)	0.496	0.89 (0.58–1.38)	0.598
Antibiotic use in the first year of life				
No	1		1	
Yes	0.75 (0.40–1.42)	0.379	1.75 (1.11–2.78)	0.017*
Exclusive breastfeeding > 4 months				
No	1		1	
Yes	1.08 (0.70–1.68)	0.717	1.43 (0.95–2.15)	0.088

Abbreviations: AD, atopic dermatitis; BMI, body mass index; CIs, confidence intervals; HR, hazard ratio.

^a^ We fitted multivariable Cox proportional hazard models to estimate hazard ratios (HRs) and 95% confidence intervals (CIs) for AD, controlling for all the factors in the table.

^b^ There is no parent‐reported AD case in this subgroup.

*P < 0.05.

The results of sensitivity analyses are similar to the main analysis. When we combined the physician‐ and parent‐reported AD diagnoses, used pooled data with multiple imputations or restricted the AD diagnoses from the age of 2, there were no statistical differences in the HR for AD development between the two cohorts (Table S1). As for the changes in risk factors over decades, when physician‐ and parent‐reported AD were combined, we found that AD development in the UBCS (2000/2001) was only associated with having first‐degree relatives with AD, and AD development in the SPATZ (2012/2013) was only associated with antibiotic use in the first year (Table S2). Pooled analysis for data with multiple imputations (Tables S3 and S4) also showed similar results for the changes of risk factors for physician‐reported AD, although antibiotic use in the first year was not statistically significant and became only marginally associated with parent‐reported AD in the 2012/2013 SPATZ cohort (HR: 1.53, 95% CI: 0.99–2.36, *p* = 0.055). When only AD diagnoses from the age of 2 were considered (Tables S5 and S6), having first‐degree relatives with AD was still the only statistically significant predictor for both physician‐ and parent‐reported AD in UBCS (2000/2001). In SPATZ (2012/2013), having at least one older sibling was significantly associated with physician‐reported AD; antibiotic use in the first year of life was significantly associated with parent‐reported AD.

## 4 Discussion

Our study provided detailed findings regarding the secular changes in the cumulative incidence of childhood AD and its risk factors between two birth cohorts in Germany, UBCS (2000/2001) and SPATZ (2012/2013), being about 12 years apart. The 4‐year cumulative incidence of both physician‐reported and parent‐reported AD remained stable over time, even after accounting for the difference in family and children's characteristics between the two cohorts. However, there was a switching pattern in the statistically significant risk factors of AD development between the 2000/2001 and the 2012/2013 birth cohorts. Compared with the 2000/2001 birth cohort, there was an attenuation of the relationship between family history of AD and childhood AD development in the 2012/2013 birth cohort, whereas having at least one older sibling and infantile antibiotic use became significant factors associated with the risk of developing AD in the 2012/2013 birth cohort. However, the limited sample size and partly overlapping CIs have to be considered. We also noted that the use of physician‐reported and parent‐reported AD as the outcomes might lead to different results regarding risk factors associated with AD development. The results of sensitivity analyses were similar to the main analysis.

Our results suggest that the cumulative prevalence of AD remained stable between the study period of the two German birth cohort studies (2000/2001–2012/2013), which is in line with previous studies on AD epidemiology in European countries [11]. While most previous studies only estimated the point prevalence or incidence of AD, our study presented cumulative incidence between the ages of 1 and 4 in the cohorts. Therefore, it can provide more informative results with multiple repeated measures and serve as better evidence regarding the secular changes of childhood AD. In contrast to the stable trend of AD development in European countries, other geographic regions, especially low‐ to middle‐income countries, have witnessed an increase in the prevalence of childhood AD over the past 30 years [3]. The process of industrialisation and increased hygiene are often regarded as important contributors to the increased prevalence of AD [12]. A possible explanation for the stable prevalence of AD in European countries is that the sound medical care system and better health promotion in these countries may offset the effect of industrialisation on the AD trend [13].

Our results also suggest that, although the trend of childhood AD was stable over time, the significant factors associated with AD development may have changed between the 2000/2001 cohort and the 2012/2013 cohort. The association between the history of first‐degree relatives' AD and both childhood physician‐reported and parent‐reported AD development was strong in UBCS (2000/2001). However, in the SPATZ cohort (2012/2013), its association with physician‐reported AD was much weaker and not statistically significant. Our previous study reported a disagreement between physician‐reported and parent‐reported childhood AD diagnoses in questionnaire‐based cohort studies [14]. We have both physician‐reported and parent‐reported AD diagnoses as outcome measures, but we could only depend on parents' questionnaires to obtain information regarding the history of first‐degree relatives' atopic status. This may have led to the insignificant association between the history of first‐degree relatives' atopic status and physicians' reported AD in SPATZ (2012/2013). The change in the strength of the association might be due to the relatively small sample size, the higher loss to follow‐up rate and the limited statistical power in the SPATZ (2012/2013) cohort. In addition, previous studies reported that the effect of genetic factors on AD development may vary by the existence of certain environmental factors (e.g., keeping a pet at home or recently moving to a newly decorated house) [15, 16]. Therefore, we cannot exclude that certain unmeasured environmental factors may have contributed to the diminished association between maternal atopic history and childhood AD development.

Besides family atopic history, having no older siblings and antibiotic use in the first year of life are two emerging risk factors for physician‐reported AD in SPATZ (2012/2013) but not in UBCS (2000/2001). While it is important to note that antibiotic exposure in the first year may be a more appropriate risk factor where AD from Year 2 is the outcome, these two factors are related to childhood microbial exposure. On the one hand, having at least one older sibling in the house will benefit children by increasing the diversity of gut microbiota [17]. On the other hand, antibiotic use in early life may alter fetal skin and gut microbiome and increase the risk of atopic diseases [18]. In the SPATZ (2012/2013), among a total of 113 participants with antibiotic prescription information at Year 1, only one prescription belonged to the common topical and systemic antibiotics choices for controlling secondary skin infection in AD management (e.g., fusidic acid, flucloxacillin, or cephalexin) [19, 20]. Thus, the association between antibiotic exposure and AD observed in the SPATZ (2012/2013) is not likely to be relevant to skin infections triggered by AD, but relevant to other infections. Our findings suggest that there might be a growing importance of environmental and microbiota‐related factors in the development of childhood AD over the decade. A study on British children aged 8–13 years showed that the association between asthma development and parental smoking was stronger in 2004–2014 compared to the early decade (1989–1999) [21]. However, to the best of our knowledge, there are no previous studies dealing with the change in the risk factors for AD development over time. Our results highlight the need for more future studies on environmental and microbiota‐related factors and potential interventions in childhood AD development.

Our study benefits from strong longitudinal data in two comparable and methodologically similar birth cohorts in the same district. One main limitation of our study is the absence of fathers' information in the analyses due to a high proportion of missing information on fathers in the SPATZ (2012/2013) cohort. We also lack some potential environmental factors for AD development, including pet ownership at home (available only for the UBCS [2000/2001]), decoration condition of the dwelling, indoor and outdoor air pollution, and the diet of mothers and children. In addition, we excluded approximately 25% of mothers recruited at baseline in the SPATZ (2012/2013) cohort as they were lost to Year 1 follow‐up, whereas the response was higher in the UBCS (2000/2001). We acknowledge the potentially reversed relationship between AD and antibiotic use, as antibiotic exposure during the first year of life can be a consequence of AD. Additionally, these two factors may be correlated through other mediating pathways potentially reflecting shared reporting biases or unmeasured confounders rather than a direct causal relationship, thereby distorting the observed association. Participants with a higher socio‐economic status and with a higher likelihood of suffering health problems may be more inclined to stay in the cohorts. It might be a source of selection bias and potentially leads to overestimating the AD incidence and missing the statistical significance of factors associated with AD. It is also possible that children with specific health conditions (e.g., lower birth weight, preterm birth, or early use of antibiotics) are more likely to be seen by physicians as well as be watched more by their parents, and therefore they are more likely to be reported as AD cases. Besides, the AD diagnosis in the two birth cohorts is not based on uniform diagnostic criteria such as the UK working party criteria [22] but rather on the report of the child's caring paediatrician. Different paediatricians could have given the answers regarding AD diagnosis based on different criteria or received notes from other specialists who were consulted by the families, for example, allergologists or dermatologists. We lacked a secondary outcome measure to examine the validation of questions used in the analysis. The sample size in our study limited the detection of trends over this time scale, but the results of our sensitivity analysis showed that stable trends of AD over the decade might be robust.

## 5 Conclusions

In conclusion, based on the comparison of longitudinal data between two birth cohorts in Germany, we found that the incidence of early childhood AD has been stable over a decade, despite possible secular changes in potential risk factors. The association between AD development and two microbiota‐related factors, that is, having at least one older sibling and infantile antibiotic use, became stronger over time; whereas the association between AD development and first‐degree relatives' history of AD was attenuated. The results regarding changes in risk factors need to be interpreted with caution due to the limited sample size, relatively high attrition rate and demographic differences between the two cohorts. Our findings contribute to the understanding of the determinants of early life AD development and highlight the need for further research to verify the changing role of environmental and microbiota‐related factors in AD development and the impact of targeted interventions.

## Author Contributions

Z.P. analyzed the data, wrote the first draft of the manuscript and edited the manuscript. D.R. was involved in the acquisition of data, data curation and project administration. H.B. assisted with project administration and resources. J.G. contributed to conceptualization, funding acquisition, project administration and resources. All the authors contributed to the result interpretation, review and manuscript editing.

## Ethics Statement

Both the UBCS and the SPATZ study were approved by the ethics board of Ulm University (UBCS: No. 98/2000; SPATZ: No. 311/11). Parents were given verbal and written informed consent before enrolment.

## Conflicts of Interest

J.G. is the project manager of and L.P.S. and Z.P. are scientists on research grants from Danone Research & Innovation to both Ulm University and Leipzig University in relation to studies of the composition of breast milk. This work is not related to the current publication.

## Supporting information


Data S1.

## Data Availability

The data that support the findings of this study are available from the corresponding author upon reasonable request.
