# Supplementary material for: Changes in Childhood Atopic Dermatitis Incidence and Risk Factors Over Time: Results From Two German Birth Cohorts
Source: Clin Exp Allergy. 2025 Apr 28;55(6):469–80. doi: 10.1111/cea.70066 (PMC12127062; doi:10.1111/cea.70066)
Supplement: Supplementary file 1 — Data S1. [file CEA-55-469-s001.docx]

**Supplementary materials**

Table S1. Sensitivity analysis on the difference in atopic dermatitis (AD) development by two cohorts

|  | UBCS (2000/2001) | SPATZ (2012/2013) | P |
| --- | --- | --- | --- |
| Model 1 |  |  |  |
| HR (95% CI) for combined AD diagnoses | 1 | 1.00 (0.81-1.23) | 0.972 |
| Model 2 |  |  |  |
| HR (95% CI) for physician-reported AD | 1 | 0.97 (0.79-1.19) | 0.790 |
| HR (95% CI) for parent-reported AD | 1 | 1.25 (0.94-1.66) | 0.118 |
| Model 3 |  |  |  |
| HR (95% CI) for physician-reported AD | 1 | 1.20 (0.91-1.58) | 0.196 |
| HR (95% CI) for parent-reported AD | 1 | 1.21 (0.86-1.71) | 0.276 |

Model 1, 2, and 3 are multivariable Cox proportional hazard models to estimate hazard ratios (HRs) and 95% confidence intervals (CIs), controlling for the maternal age at baseline, mothers’ education level, children’s first-degree relatives’ history of AD at baseline, mother ever having a fever during pregnancy, maternal smoking status during pregnancy, maternal BMI before pregnancy, children’s sex, preterm birth, delivery mode, birth weight, exclusive breastfeeding duration, history of antibiotic use in the first year of life, and having at least one older sibling or not.

Model 1: use combined physician- and parent-reported AD as the outcome (any AD diagnosis reported vs no AD diagnosis reported in physicians’ and parents’ questionnaires)

Model 2: use data with multiple imputations. We generated 20 imputed datasets with a fully conditional specification approach, using all the covariates above as predictors for missing values. The pooled results are shown.

Model 3: use AD diagnosis from the age of 2 as the outcome

Abbreviations: HR: hazard ratio, CIs: confidence intervals, AD: atopic dermatitis, BMI: body mass index

Table S2. Risk factors associated with combined physician- and parent-reported AD in UBCS and SPATZ cohort.

|  | **UBCS (2000/2001)** | | **SPATZ (2012/2013)** | |
| --- | --- | --- | --- | --- |
|  | HR (95% CIs)^1^ | P | HR (95% CIs) | P |
| **Family characteristics** |  |  |  |  |
| Maternal age during pregnancy |  |  |  |  |
| ≥30 years old & <35 years old | 1 |  | 1 |  |
| <30 years old | 0.98 (0.70-1.37) | 0.893 | 1.38 (0.94-2.01) | 0.098 |
| ≥35 years old | 0.81 (0.59-1.13) | 0.218 | 1.04 (0.73-1.50) | 0.813 |
| Maternal schooling >11 years |  |  |  |  |
| No | 1 |  | 1 |  |
| Yes | 1.03 (0.78-1.37) | 0.820 | 1.10 (0.78-1.54) | 0.597 |
| First-degree relatives with AD |  |  |  |  |
| No | 1 |  | 1 |  |
| Yes | 1.71 (1.24-2.37) | 0.001* | 1.39 (0.98-1.98) | 0.065 |
| Maternal BMI>25 |  |  |  |  |
| No | 1 |  | 1 |  |
| Yes | 0.94 (0.69-1.29) | 0.690 | 0.79 (0.56-1.12) | 0.185 |
| Mothers having a fever during pregnancy |  |  |  |  |
| No | 1 |  | 1 |  |
| Yes | 1.36 (0.99-1.85) | 0.055 | 0.98 (0.59-1.61) | 0.925 |
| Maternal smoking during pregnancy |  |  |  |  |
| No | 1 |  | 1 |  |
| Yes | 1.36 (0.82-2.24) | 0.234 | 1.03 (0.44-2.39) | 0.945 |
| **Children’s characteristics** |  |  |  |  |
| Sex |  |  |  |  |
| Female | 1 |  | 1 |  |
| Male | 0.87 (0.66-1.14) | 0.302 | 1.07 (0.79-1.45) | 0.656 |
| Preterm birth |  |  |  |  |
| No | 1 |  | 1 |  |
| Yes | 0.87 (0.42-1.80) | 0.701 | 0.74 (0.39-1.44) | 0.380 |
| C-section delivery |  |  |  |  |
| No | 1 |  | 1 |  |
| Yes | 1.07 (0.74-1.56) | 0.722 | 1.10 (0.78-1.55) | 0.576 |
| Birth weight |  |  |  |  |
| ≥2500g & <4000g | 1 |  | 1 |  |
| <2500g | 0.31 (0.07-1.26) | 0.100 | 0.93 (0.43-2.01) | 0.858 |
| ≥4000g | 1.17 (0.79-1.73) | 0.442 | 0.89 (0.50-1.59) | 0.700 |
| Having at least one older sibling |  |  |  |  |
| No | 1 |  | 1 |  |
| Yes | 1.04 (0.78-1.39) | 0.787 | 0.73 (0.53-1.01) | 0.056 |
| Antibiotic use in the first year of life |  |  |  |  |
| No | 1 |  | 1 |  |
| Yes | 1.09 (0.75-1.58) | 0.663 | 1.56 (1.09-2.21) | 0.014* |
| Exclusive breastfeeding >4 months |  |  |  |  |
| No | 1 |  | 1 |  |
| Yes | 1.04 (0.78-1.39) | 0.782 | 1.19 (0.89-1.60) | 0.249 |

1. We fitted multivariable Cox proportional hazard models to estimate hazard ratios (HRs) and 95% confidence intervals (CIs) for combined physician- and parent-reported AD (any AD diagnosis reported vs no AD diagnosis reported in physicians’ and parents’ questionnaires), controlling for all the factors in the table.

*： P<0.05

Abbreviations: HR: hazard ratio, CIs: confidence intervals, BMI: body mass index; AD: atopic dermatitis

Table S3. Risk factors associated with physician-reported AD in the UBCS and SPATZ cohort, using data with multiple imputations

|  | **UBCS (2000/2001)** | | **SPATZ (2012/2013)** | |
| --- | --- | --- | --- | --- |
|  | HR (95% CIs)^1^ | P | HR (95% CIs) | P |
| **Family characteristics** |  |  |  |  |
| Maternal age during pregnancy |  |  |  |  |
| ≥30 years old & <35 years old | 1 |  | 1 |  |
| <30 years old | 1.06 (0.78-1.43) | 0.730 | 1.26 (0.85-1.86) | 0.251 |
| ≥35 years old | 1.06 (0.77-1.47) | 0.702 | 1.07 (0.74-1.55) | 0.728 |
| Maternal schooling >11 years |  |  |  |  |
| No | 1 |  | 1 |  |
| Yes | 0.87 (0.65-1.17) | 0.358 | 1.01 (0.72-1.41) | 0.970 |
| First-degree relatives with AD |  |  |  |  |
| No | 1 |  | 1 |  |
| Yes | 1.62 (1.18-2.24) | 0.003* | 1.12 (0.74-1.69) | 0.591 |
| Maternal BMI>25 |  |  |  |  |
| No | 1 |  | 1 |  |
| Yes | 0.95 (0.71-1.28) | 0.748 | 0.81 (0.58-1.14) | 0.232 |
| Mothers having a fever during pregnancy |  |  |  |  |
| No | 1 |  | 1 |  |
| Yes | 1.16 (0.85-1.57) | 0.342 | 1.19 (0.71-1.99) | 0.516 |
| Maternal smoking during pregnancy |  |  |  |  |
| No | 1 |  | 1 |  |
| Yes | 0.96 (0.64-1.45) | 0.857 | 0.97 (0.41-2.33) | 0.947 |
| **Children’s characteristics** |  |  |  |  |
| Sex |  |  |  |  |
| Female | 1 |  | 1 |  |
| Male | 1.03 (0.79-1.35) | 0.811 | 1.13 (0.81-1.58) | 0.464 |
| Preterm birth |  |  |  |  |
| No | 1 |  | 1 |  |
| Yes | 0.65 (0.31-1.33) | 0.237 | 0.53 (0.25-1.12) | 0.098 |
| C-section delivery |  |  |  |  |
| No | 1 |  | 1 |  |
| Yes | 1.19 (0.84-1.69) | 0.316 | 1.18 (0.81-1.70) | 0.385 |
| Birth weight |  |  |  |  |
| ≥2500g & <4000g | 1 |  | 1 |  |
| <2500g | 0.40 (0.12-1.35) | 0.318 | 0.77 (0.35-1.69) | 0.513 |
| ≥4000g | 0.88 (0.58-1.33) | 0.542 | 0.94 (0.54-1.65) | 0.826 |
| Having at least one older sibling |  |  |  |  |
| No | 1 |  | 1 |  |
| Yes | 0.90 (0.67-1.21) | 0.486 | 0.73 (0.52-1.02) | 0.068 |
| Antibiotic use in the first year of life |  |  |  |  |
| No | 1 |  | 1 |  |
| Yes | 1.22 (0.85-1.76) | 0.277 | 1.44 (1.00-2.05) | 0.047* |
| Exclusive breastfeeding >4 months |  |  |  |  |
| No | 1 |  | 1 |  |
| Yes | 0.97 (0.74-1.28) | 0.837 | 1.16 (0.85-1.59) | 0.337 |

1. We generated 20 imputed datasets with a fully conditional specification approach, using all the covariates above as predictors for missing values. Then we fitted multivariable Cox proportional hazard models to estimate hazard ratios (HRs) and 95% confidence intervals (CIs) for AD, controlling for all the factors in the table. The pooled results are shown.

*： P<0.05

Abbreviations: HR: hazard ratio, CIs: confidence intervals, BMI: body mass index; AD: atopic dermatitis

Table S4. Risk factors associated with parent-reported AD in the UBCS and SPATZ cohort, using data with multiple imputations

|  | **UBCS (2000/2001)** | | **SPATZ (2012/2013)** | |
| --- | --- | --- | --- | --- |
|  | HR (95% CIs)^1^ | P | HR (95% CIs) | P |
| **Family characteristics** |  |  |  |  |
| Maternal age during pregnancy |  |  |  |  |
| ≥30 years old & <35 years old | 1 |  | 1 |  |
| <30 years old | 0.87 (0.56-1.34) | 0.514 | 1.36 (0.83-2.23) | 0.219 |
| ≥35 years old | 0.98 (0.64-1.50) | 0.933 | 1.23 (0.78-1.93) | 0.375 |
| Maternal schooling >11 years |  |  |  |  |
| No | 1 |  | 1 |  |
| Yes | 1.09 (0.75-1.58) | 0.663 | 1.10 (0.74-1.64) | 0.633 |
| First-degree relatives with AD |  |  |  |  |
| No | 1 |  | 1 |  |
| Yes | 1.80 (1.20-2.71) | 0.005* | 1.56 (0.99-2.45) | 0.057 |
| Maternal BMI>25 |  |  |  |  |
| No | 1 |  | 1 |  |
| Yes | 0.72 (0.47-1.10) | 0.128 | 0.91 (0.60-1.38) | 0.651 |
| Mothers having a fever during pregnancy |  |  |  |  |
| No | 1 |  | 1 |  |
| Yes | 1.13 (0.75-1.72) | 0.555 | 0.84 (0.44-1.61) | 0.604 |
| Maternal smoking during pregnancy |  |  |  |  |
| No | 1 |  | 1 |  |
| Yes | 1.58 (0.93-2.69) | 0.094 | 1.83 (0.92-3.67) | 0.087 |
| **Children’s characteristics** |  |  |  |  |
| Sex |  |  |  |  |
| Female | 1 |  | 1 |  |
| Male | 0.93 (0.65-1.32) | 0.682 | 1.22 (0.85-1.75) | 0.289 |
| Preterm birth |  |  |  |  |
| No | 1 |  | 1 |  |
| Yes | 0.56 (0.17-1.84) | 0.334 | 0.54 (0.22-1.34) | 0.186 |
| C-section delivery |  |  |  |  |
| No | 1 |  | 1 |  |
| Yes | 0.97 (0.60-1.59) | 0.918 | 0.89 (0.56-1.41) | 0.622 |
| Birth weight |  |  |  |  |
| ≥2500g & <4000g | 1 |  | 1 |  |
| <2500g | N/A ^2^ | N/A | 1.12 (0.46-2.74) | 0.807 |
| ≥4000g | 1.12 (0.64-1.94) | 0.694 | 1.41 (0.74-2.66) | 0.292 |
| Having at least one older sibling |  |  |  |  |
| No | 1 |  | 1 |  |
| Yes | 0.98 (0.65-1.46) | 0.906 | 0.92 (0.62-1.38) | 0.690 |
| Antibiotic use in the first year of life |  |  |  |  |
| No | 1 |  | 1 |  |
| Yes | 0.98 (0.58-1.65) | 0.929 | 1.53 (0.99-2.36) | 0.055 |
| Exclusive breastfeeding >4 months |  |  |  |  |
| No | 1 |  | 1 |  |
| Yes | 1.12 (0.78-1.62) | 0.531 | 1.41 (0.95-2.08) | 0.087 |

1. We generated 20 imputed datasets with a fully conditional specification approach, using all the covariates above as predictors for missing values. Then we fitted multivariable Cox proportional hazard models to estimate hazard ratios (HRs) and 95% confidence intervals (CIs) for AD, controlling for all the factors in the table. The pooled results are shown.
2. There is no parent-reported AD case in this subgroup in the original data.

*： P<0.05

Abbreviations: HR: hazard ratio, CIs: confidence intervals, BMI: body mass index; AD: atopic dermatitis

Table S5. Risk factors associated with physician-reported AD from the age of 2 in the UBCS and SPATZ cohort.

|  | **UBCS (2000/2001)** | | **SPATZ (2012/2013)** | |
| --- | --- | --- | --- | --- |
|  | HR (95% CIs)^1^ | P | HR (95% CIs) | P |
| **Family characteristics** |  |  |  |  |
| Maternal age during pregnancy |  |  |  |  |
| ≥30 years old & <35 years old | 1 |  | 1 |  |
| <30 years old | 0.86 (0.55-1.35) | 0.508 | 1.55 (0.97-2.48) | 0.069 |
| ≥35 years old | 1.00 (0.66-1.53) | 0.985 | 1.27 (0.82-1.98) | 0.283 |
| Maternal schooling >11 years |  |  |  |  |
| No | 1 |  | 1 |  |
| Yes | 0.86 (0.59-1.24) | 0.410 | 0.96 (0.63-1.46) | 0.852 |
| First-degree relatives with AD |  |  |  |  |
| No | 1 |  | 1 |  |
| Yes | 1.68 (1.10-2.55) | 0.016* | 1.12 (0.71-1.79) | 0.624 |
| Maternal BMI>25 |  |  |  |  |
| No | 1 |  | 1 |  |
| Yes | 0.95 (0.63-1.43) | 0.791 | 0.77 (0.50-1.18) | 0.232 |
| Mothers having a fever during pregnancy |  |  |  |  |
| No | 1 |  | 1 |  |
| Yes | 0.91 (0.58-1.43) | 0.690 | 1.29 (0.72-2.31) | 0.385 |
| Maternal smoking during pregnancy |  |  |  |  |
| No | 1 |  | 1 |  |
| Yes | 0.97 (0.48-1.95) | 0.920 | 0.63 (0.19-2.04) | 0.435 |
| **Children’s characteristics** |  |  |  |  |
| Sex |  |  |  |  |
| Female | 1 |  | 1 |  |
| Male | 0.83 (0.58-1.18) | 0.289 | 1.05 (0.72-1.53) | 0.802 |
| Preterm birth |  |  |  |  |
| No | 1 |  | 1 |  |
| Yes | 0.99 (0.39-2.53) | 0.989 | 0.51 (0.21-1.25) | 0.142 |
| C-section delivery |  |  |  |  |
| No | 1 |  | 1 |  |
| Yes | 0.87 (0.53-1.45) | 0.602 | 1.16 (0.76-1.78) | 0.487 |
| Birth weight |  |  |  |  |
| ≥2500g & <4000g | 1 |  | 1 |  |
| <2500g | 0.31 (0.04-2.33) | 0.257 | 1.27 (0.52-3.10) | 0.608 |
| ≥4000g | 1.02 (0.59-1.77) | 0.936 | 0.91 (0.45-1.85) | 0.798 |
| Having at least one older sibling |  |  |  |  |
| No | 1 |  | 1 |  |
| Yes | 0.97 (0.66-1.42) | 0.869 | 0.57 (0.38-0.86) | 0.007* |
| Antibiotic use in the first year of life |  |  |  |  |
| No | 1 |  | 1 |  |
| Yes | 0.76 (0.43-1.34) | 0.348 | 1.28 (0.80-2.03) | 0.308 |
| Exclusive breastfeeding >4 months |  |  |  |  |
| No | 1 |  | 1 |  |
| Yes | 0.82 (0.56-1.20) | 0.303 | 1.20 (0.82-1.72) | 0.355 |

1. We fitted multivariable Cox proportional hazard models to estimate hazard ratios (HRs) and 95% confidence intervals (CIs) for AD, controlling for all the factors in the table.

*： P<0.05

Abbreviations: HR: hazard ratio, CIs: confidence intervals, BMI: body mass index; AD: atopic dermatitis

Table S6. Risk factors associated with parent-reported AD from the age of 2 in the UBCS and SPATZ cohort.

|  | **UBCS (2000/2001)** | | **SPATZ (2012/2013)** | |
| --- | --- | --- | --- | --- |
|  | HR (95% CIs)^1^ | P | HR (95% CIs) | P |
| **Family characteristics** |  |  |  |  |
| Maternal age during pregnancy |  |  |  |  |
| ≥30 years old & <35 years old | 1 |  | 1 |  |
| <30 years old | 1.00 (0.58-1.74) | 0.993 | 2.16 (1.18-3.94) | 0.012* |
| ≥35 years old | 0.77 (0.44-1.33) | 0.344 | 1.50 (0.84-2.66) | 0.167 |
| Maternal schooling >11 years |  |  |  |  |
| No | 1 |  | 1 |  |
| Yes | 1.10 (0.69-1.75) | 0.702 | 1.08 (0.63-1.85) | 0.784 |
| First-degree relatives with AD |  |  |  |  |
| No | 1 |  | 1 |  |
| Yes | 2.14 (1.29-3.54) | 0.003* | 1.55 (0.91-2.65) | 0.110 |
| Maternal BMI>25 |  |  |  |  |
| No | 1 |  | 1 |  |
| Yes | 0.83 (0.48-1.44) | 0.502 | 0.72 (0.41-1.25) | 0.242 |
| Mothers having a fever during pregnancy |  |  |  |  |
| No | 1 |  | 1 |  |
| Yes | 0.98 (0.56-1.70) | 0.935 | 0.86 (0.38-1.90) | 0.701 |
| Maternal smoking during pregnancy |  |  |  |  |
| No | 1 |  | 1 |  |
| Yes | 1.31 (0.55-3.11) | 0.541 | 1.22 (0.36-4.06) | 0.750 |
| **Children’s characteristics** |  |  |  |  |
| Sex |  |  |  |  |
| Female | 1 |  | 1 |  |
| Male | 0.94 (0.60-1.46) | 0.771 | 1.10 (0.68-1.78) | 0.692 |
| Preterm birth |  |  |  |  |
| No | 1 |  | 1 |  |
| Yes | 0.96 (0.29-3.11) | 0.940 | 0.40 (0.12-1.26) | 0.116 |
| C-section delivery |  |  |  |  |
| No | 1 |  | 1 |  |
| Yes | 0.91 (0.46-1.79) | 0.781 | 0.88 (0.50-1.54) | 0.652 |
| Birth weight |  |  |  |  |
| ≥2500g & <4000g | 1 |  | 1 |  |
| <2500g | N/A ^2^ | N/A | 2.54 (0.88-7.39) | 0.086 |
| ≥4000g | 0.95 (0.47-1.93) | 0.890 | 1.22 (0.54-2.74) | 0.639 |
| Having at least one older sibling |  |  |  |  |
| No | 1 |  | 1 |  |
| Yes | 1.08 (0.66-1.75) | 0.764 | 0.91 (0.55-1.49) | 0.694 |
| Antibiotic use in the first year of life |  |  |  |  |
| No | 1 |  | 1 |  |
| Yes | 0.68 (0.32-1.42) | 0.300 | 2.10 (1.25-3.52) | 0.005* |
| Exclusive breastfeeding >4 months |  |  |  |  |
| No | 1 |  | 1 |  |
| Yes | 1.18 (0.72-1.94) | 0.513 | 1.48 (0.92-2.37) | 0.103 |

1. We fitted multivariable Cox proportional hazard models to estimate hazard ratios (HRs) and 95% confidence intervals (CIs) for AD, controlling for all the factors in the table.
2. There is no parent-reported AD case in this subgroup

*： P<0.05

Abbreviations: HR: hazard ratio, CIs: confidence intervals, BMI: body mass index; AD: atopic dermatitis
